# Author Correction: Involvement of G-quadruplex regions in mammalian replication origin activity

**DOI:** 10.1038/s41467-020-16122-x

**Published:** 2020-06-11

**Authors:** Paulina Prorok, Marie Artufel, Antoine Aze, Philippe Coulombe, Isabelle Peiffer, Laurent Lacroix, Aurore Guédin, Jean-Louis Mergny, Julia Damaschke, Aloys Schepers, Christelle Cayrou, Marie-Paule Teulade-Fichou, Benoit Ballester, Marcel Méchali

**Affiliations:** 1 Institute of Human Genetics, CNRS-University of Montpellier, 141 rue de la Cardonille, 34396 Montpellier, France; 2 Aix Marseille Univ, INSERM, TAGC, Marseille, France; 3 Balasubramanian group, Department of Chemistry, University of Cambridge, Lensfield Road, Cambridge, CB2 1EW UK; 4 ARNA Laboratory, Université de Bordeaux, Inserm U1212, CNRS UMR5320, Institut Européen de Chimie Biologie (IECB), Pessac, 33607 France; 5 Institut Curie, CNRS UMR9187, Inserm U1196, Universite Paris Saclay, Orsay, France; 6 Research Unit Gene Vectors, Helmholtz Zentrum München (GmbH), German Research Center for Environmental Health, Marchioninistraße 25, 81377 Munich, Germany; 7 Monoclonal Antibody Core Facility & Research Group, Institute for Diabetes and Obesity, Helmholtz Zentrum München, Ingolstädter Landstrasse, 85764 Neuherberg, Germany; 8 CMIB Laboratory, Institut Curie, Campus Universitaire, Bat, 110 91405 Orsay, France; 9 Present Address: Centre de Recherche en Cancérologie de Marseille 27 Boulevard Lei Roure, 13273 Marseille, France

**Keywords:** Molecular biology, Origin selection, Chromatin

Correction to: *Nature Communications* 10.1038/s41467-019-11104-0, published online 22 July 2019.

The original version of this Article omitted the authors Christelle Cayrou from the Centre de Recherche en Cancérologie de Marseille, 27 Boulevard Lei Roure, 13273 Marseille, France; and Marie-Paule Teulade-Fichou, from CMIB Laboratory, Institut Curie, Campus Universitaire, Bat 110 91405 Orsay, France.

Additionally, the following were originally omitted from the Acknowledgements: ‘the authors thank Stéphane Bocquet (IGH) for help with Xenopus egg extracts preparation’, and ‘This work was supported by the Association Française contre les Myopathies (AFM)’.The corrected version of the Acknowledgements also removes the following from the original version: ‘We thank Marie-Paule Teulade-Fichou (Institut Curie, Orsay, France) for providing us PhenDC3’.

Additionally, the following was originally omitted from the Author Contributions: ‘C.C. proposed the experimental system and the design for the experiments involving Phen DC, and M.P.F.T. provided PhenDC3 and helped in the use of this drug’.

This has been corrected in both the PDF and HTML versions of the Article.

