# Supplementary material for: Involvement of G-quadruplex regions in mammalian replication origin activity
Source: Nat Commun. 2019 Jul 22;10:3274. doi: 10.1038/s41467-019-11104-0 (PMC6646384; doi:10.1038/s41467-019-11104-0)
Supplement: Supplementary file 1 — Supplementary Information [file 41467_2019_11104_MOESM1_ESM.pdf]

Involvement of G-quadruplex regions in mammalian replication origin activity

Prorok et al.

Supplementary Information

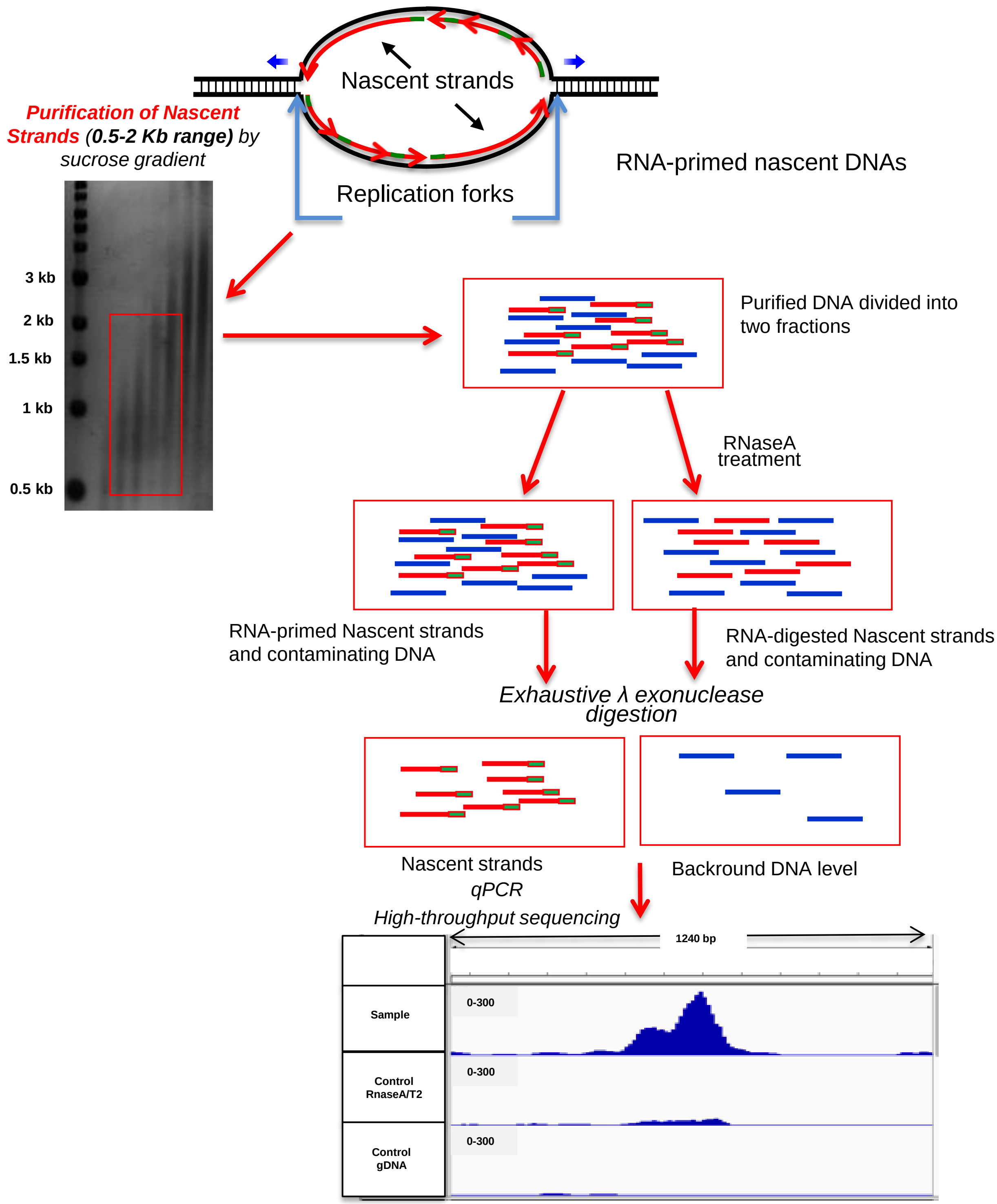

Supplementary Figure 1. DNA replication origin mapping by nascent DNA strand isolation.

Short nascent DNA strands (0.5-2 kb) were isolated by genomic DNA purification and denaturation followed by nascent strand isolation on sucrose gradients. The nascent strand population was then digested with λ exonuclease, as described in Cayrou et al, Gen Res 2015 and Methods. The background level that might be left after λ exonuclease digestion was measured by treating half of the nascent DNA strand sample with RNase A/RNase T2 prior to a second round of λ exonuclease digestion. Purified nascent strands were then analyzed by qPCR or high-throughput whole-genome sequencing.

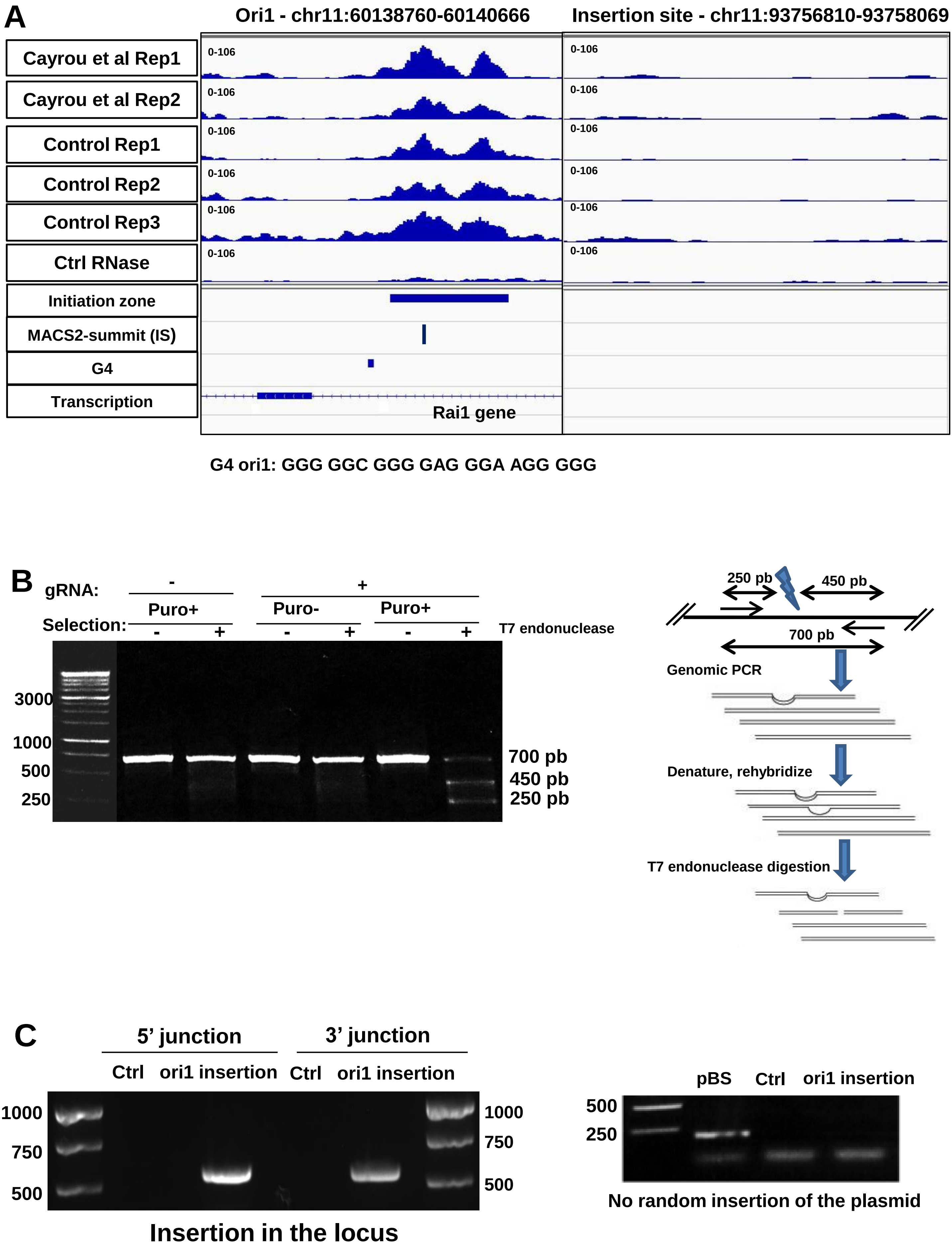
A
Ori1 - chr11:60138760-60140666
Insertion site - chr11:93756810-93758069
Cayrou et al Rep1
Cayrou et al Rep2
Control Rep1
Control Rep2
Control Rep3
Ctrl RNase
Initiation zone
MACS2-summit (IS)
G4
Transcription
0-106
Rai1 gene
G4 ori1: GGG GGC GGG GAG GGA AGG GGG
B
gRNA:
-
+
Selection:
Puro+
Puro-
Puro+
T7 endonuclease
3000
1000
500
250
700 pb
450 pb
250 pb
250 pb
450 pb
700 pb
Genomic PCR
Denature, rehybridize
T7 endonuclease digestion
C
5' junction
3' junction
Ctrl
ori1 insertion
1000
750
500
Insertion in the locus
pBS
Ctrl
ori1 insertion
500
250
No random insertion of the plasmid

Supplementary Figure 2. Characterization of the ectopic replication origin.

A) Replication profile of the origin site (Ori1) used for this study, in five entirely independent replicates of mouse ES cells (two from data in in Cayrou et al, Gen Res 2015 and three from the present analyses left panel). The selected origin on chromosome 11 is associated with a putative OGRE/G4-forming sequence, used in the presented study, that is located 240 bp upstream of the IS, defined by MACS2-summit, the point having the maximum reads number scored within the initiation zone. The ectopic Ori1 insertion site (right panel) is situated in a large origin-, transcription-, and OGRE/G4-free region.

B) Ori1 was inserted in the ectopic site by homologous recombination between the linear recombination template and the targeted site in the genome that was stimulated by a double strand break (DSB) induced by the Cas9 endonuclease expressed in transfected cells (right scheme). The specificity of DSB formation by Cas9 was checked using the Surveyor assay (left panel). The T7 endonuclease cuts mismatched regions in the dsDNA. A DSB was created at the targeted site only in the presence of a gRNA, and when puromycin was used for selection of transfected cells.

C) The correct ectopic insertion in the selected locus was confirmed by PCR detection of the newly created 5’ and 3’ junctions in the genome (left panel). The absence of any random insertion of the pBluescript plasmid bearing the insert was confirmed using primers specific for the plasmid (right panel).

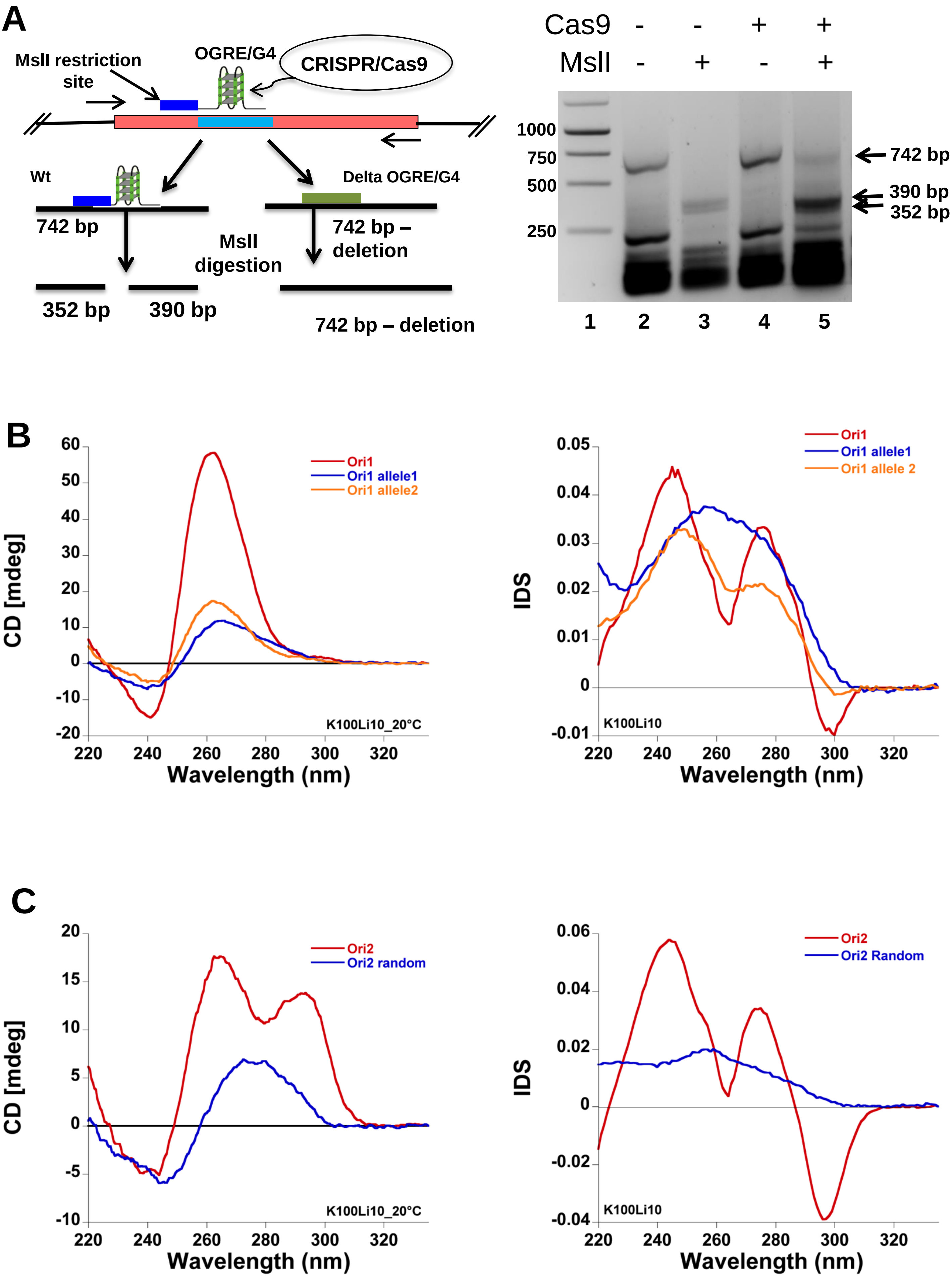
A
MslI restriction site
OGRE/G4
CRISPR/Cas9
Wt
Delta OGRE/G4
742 bp
MslI digestion
742 bp – deletion
352 bp
390 bp
742 bp – deletion
Cas9 - - + +
MslI - + - +
1000
750
500
250
1 2 3 4 5
742 bp
390 bp
352 bp
B
CD [mdeg]
Ori1
Ori1 allele1
Ori1 allele2
K100Li10_20°C
Wavelength (nm)
IDS
Ori1
Ori1 allele1
Ori1 allele 2
K100Li10
Wavelength (nm)
C
CD [mdeg]
Ori2
Ori2 random
K100Li10_20°C
Wavelength (nm)
IDS
Ori2
Ori2 Random
K100Li10
Wavelength (nm)

Supplementary Figure 3. Characterization of the G4 deletion from an endogenous replication origin.

A) Schematic description of the OGRE/G4-containing Ori1 sequence deletion procedure (left panel). Sequence deletion was confirmed by digestion with MslI (a restriction enzyme that recognizes a palindromic sequence close to the OGRE/G4 motif). The DNA fragment with the targeted region (742 bp) was amplified by PCR and digested with MslI (right panel). As the deletion abolishes this site, a non-cleaved product is observed. In the wild type allele, incubation with MslI leads to the formation of two fragments (352 bp and 390 bp). The cell population harboring the deletion was cloned and in each clone, the presence of the deletion was confirmed by sequencing.

B) Circular dichroism spectra (CD, right panel) and Isothermal differential spectra (IDS, left panel) of Ori1 and muateted alleles obtained during CRISPR-Cas9 driven G4 deletion. Strong hallmarks of G4 formation in the wt sequence (Ori1), including a strong positive peak around 260 nm on CD spectrum, and a strong negative peak around 295 nm accompanied by two positive peaks around 240 and 273 nm on IDS spectrum are lost upon G4 deletion (Ori1 allele 1, Ori1 allele 2). The sequences were tested at 4 µM strand concentration in 10 mM LiCaco pH 7,2 with 100 mM K+; CD spectra were recorded at 20°C after UV melting (in K^+) between the 220–335 nm on a JASCO-1500 spectropolarimeter using 1 cm path length quartz cuvettes. IDS were obtained by taking the difference between the absorbance spectra from unfolded (without K^+) and folded oligonucleotides (with K^+). These spectra were respectively recorded before and after potassium cation addition at 25°C. They were recorded between the 220–335 nm.

C) Circular dichroism spectra (CD; right panel) and Isothermal differential spectra (IDS; left panel) of Ori2 and randomized Ori2. Hallmarks of G4 formation (a strong positive peak around 260 nm on CD spectrum, and a strong negative peak around 295 nm together with two positive peaks around 240 and 273 nm on IDS spectrum) present in the wt sequence (Ori2) are lost upon G4 randomisation (Ori2 Random).

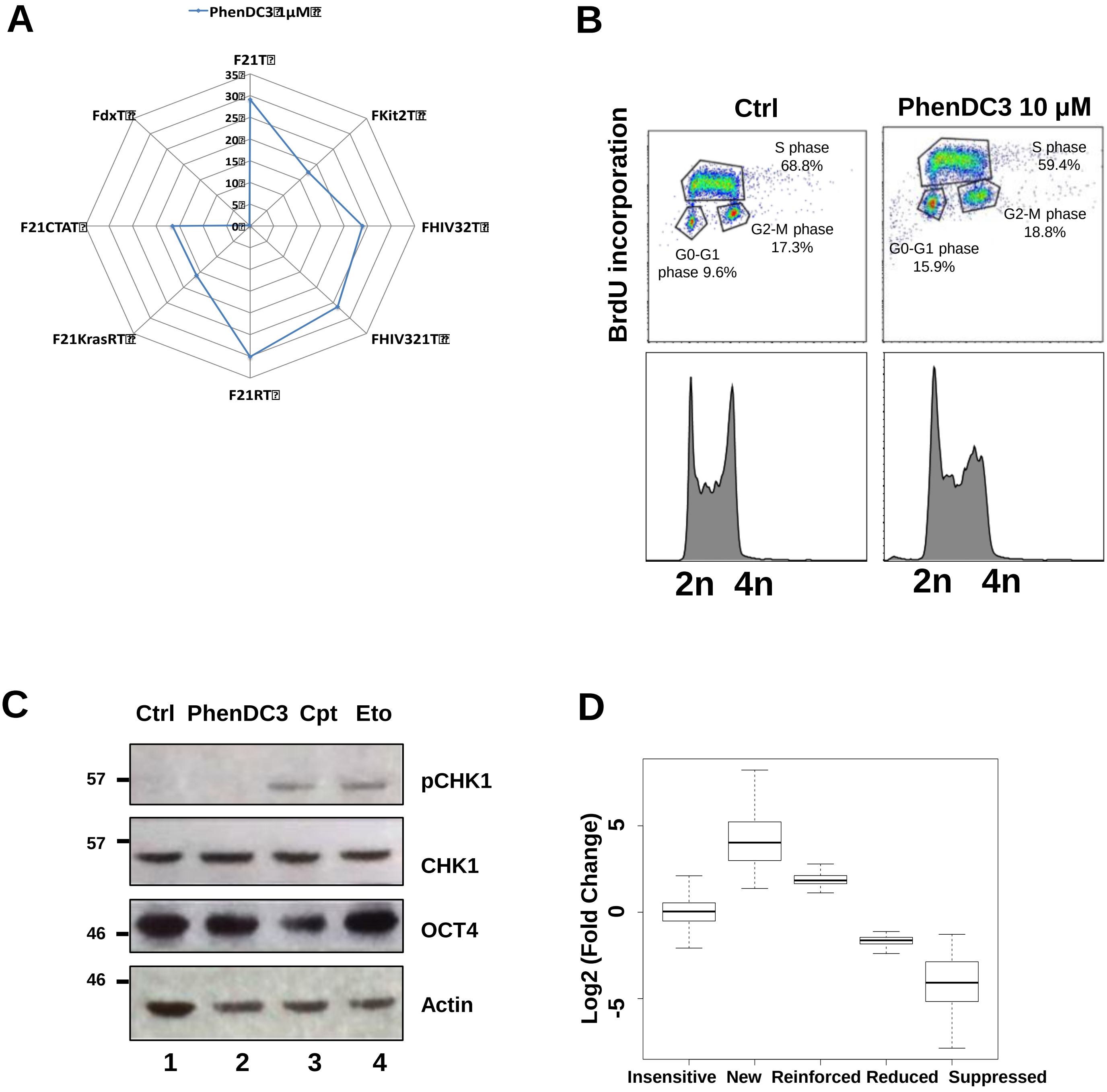
A
PhenDC3 1µM
F21T
35
30
25
20
15
10
5
0
FKit2T
FHIV32T
FHIV321T
F21RT
F21KrasRT
F21CTAT
FdxT
B
Ctrl
PhenDC3 10 µM
BrdU incorporation
S phase 68.8%
G2-M phase 17.3%
G0-G1 phase 9.6%
S phase 59.4%
G2-M phase 18.8%
G0-G1 phase 15.9%
2n 4n
2n 4n
C
Ctrl PhenDC3 Cpt Eto
57
57
46
46
pCHK1
CHK1
OCT4
Actin
1 2 3 4
D
Log2 (Fold Change)
5
0
-5
Insensitive New Reinforced Reduced Suppressed

Supplementary Figure 4. PhenDC3 is an ubiquitous G4-forming sequence ligand.

A) FRET melting assay: PhenDC3 stabilization of several G4-forming sequences labeled with Fam on the 5’ and Tamra on the 3’. Each G4-forming sequence was pre-folded at 0.2 µM in 10 mM LiCaco pH 7.2 supplemented with 10 mM KCl and 90 mM LiCl before adding 1 µM PhenDC3. Stabilization (i.e., increase in $T_{1/2}$, expressed in °C) is plotted for seven different quadruplexes and one control duplex (FdxT). The means were obtained in two independent expreiments ± SD. The tested sequences were: F21T (human telomeric DNA), F21RT (human telomeric RNA), Fkit2T (c-KIT2 human oncogene promoter), F21CTAT (mutant human telomeric DNA), FHiv32T (HIV PRO2 sequence), FHiv321T (HIV PRO1 sequence) and FdxT (intramolecular duplex). PRO1 and PRO2 are two different parts of the same HIV promoter region. The sequences are provided in Supplemental Table 3.

B) The cell cycle is not perturbed by incubation with PhenDC3, as indicated by the similar FACS profiles of control ES cells and cells incubated with 10 µM PhenDC3.

C) G4 stabilization does not induce checkpoint activity, differently from camptothecin (Cpt) and etoposide (Eto), two known checkpoint-inducing agents. Expression of the pluripotency marker OCT4 did not vary after PhenDC3 addition (for antibody references see Supplementary Table 7).

D) The fold change in read number after incubation with PhenDC3 compared with control was used to define the different origin classes. No change in read number was observed for origins in the insensitive class. The highest positive fold change was observed in the new class, followed by the reinforced class. The most negative fold change was in the suppressed class.

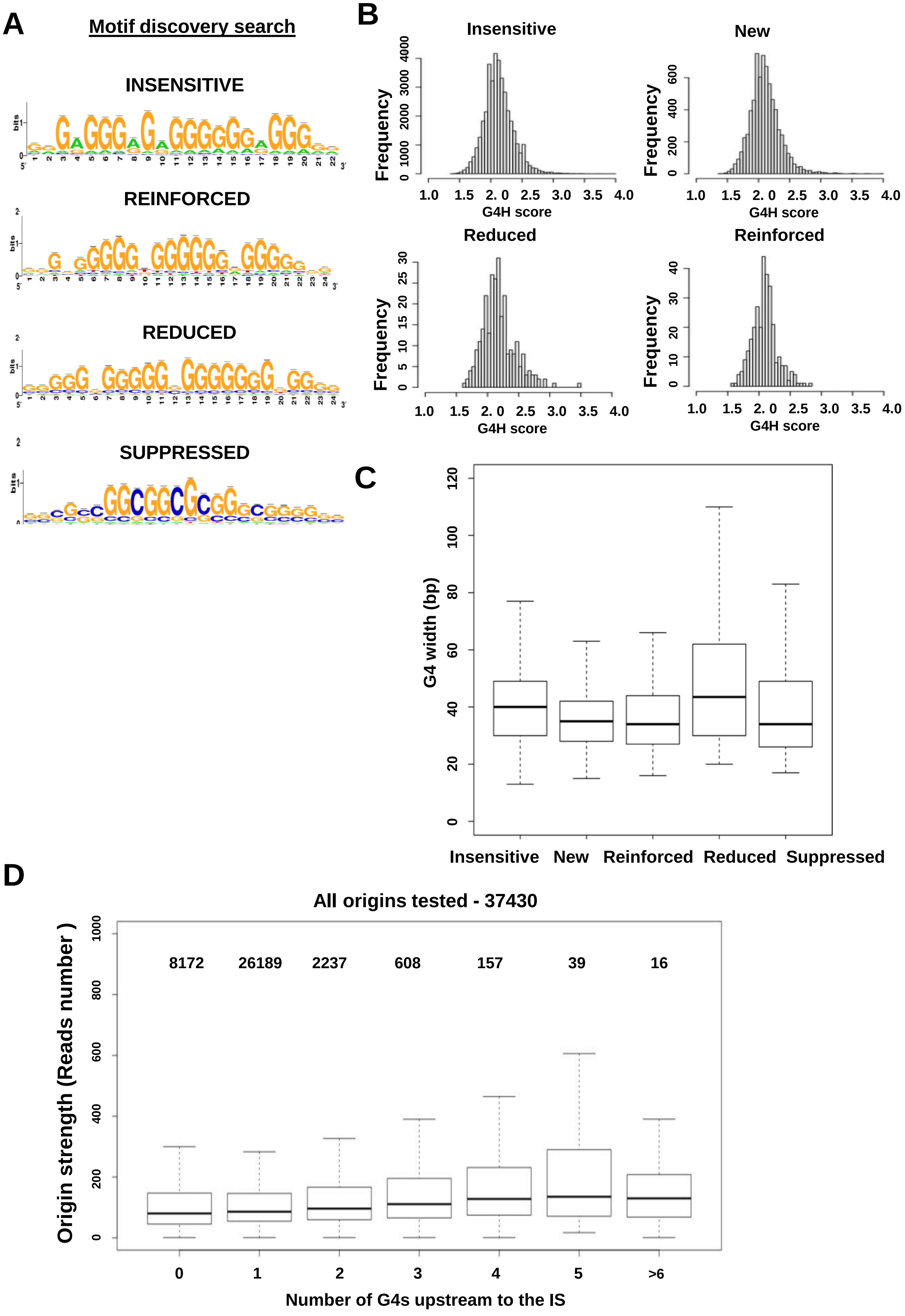
A
Motif discovery search
INSENSITIVE
REINFORCED
REDUCED
SUPPRESSED
B
Insensitive
New
Reduced
Reinforced
Frequency
G4H score
C
G4 width (bp)
Insensitive
New
Reinforced
Reduced
Suppressed
D
All origins tested - 37430
8172
26189
2237
608
157
39
16
Origin strength (Reads number)
0
1
2
3
4
5
>6
Number of G4s upstream to the IS

Supplementary Figure 5. OGRE/G4 features in the different origin classes after G4 stabilization by PhenDC3.

A) De novo motif discovery for each origin class revealed the presence of an OGRE/G4 sequence, but for the suppressed class (analysis preformed using the RSAT suite [57] and [4]. For the motif found in the new origin class see Figure 4A.

B) Distribution of the G4-Hunter (G4H) score is similar in all G4-associated origin classes (*reduced, reinforced, new, and insensitive*).

C) The length of the OGRE/G4 sequence does not significantly vary in the different origin classes.

D) Replication origin strength (measured in reads number) increases slightly with the number of OGRE/G4 associated with the tested origin.

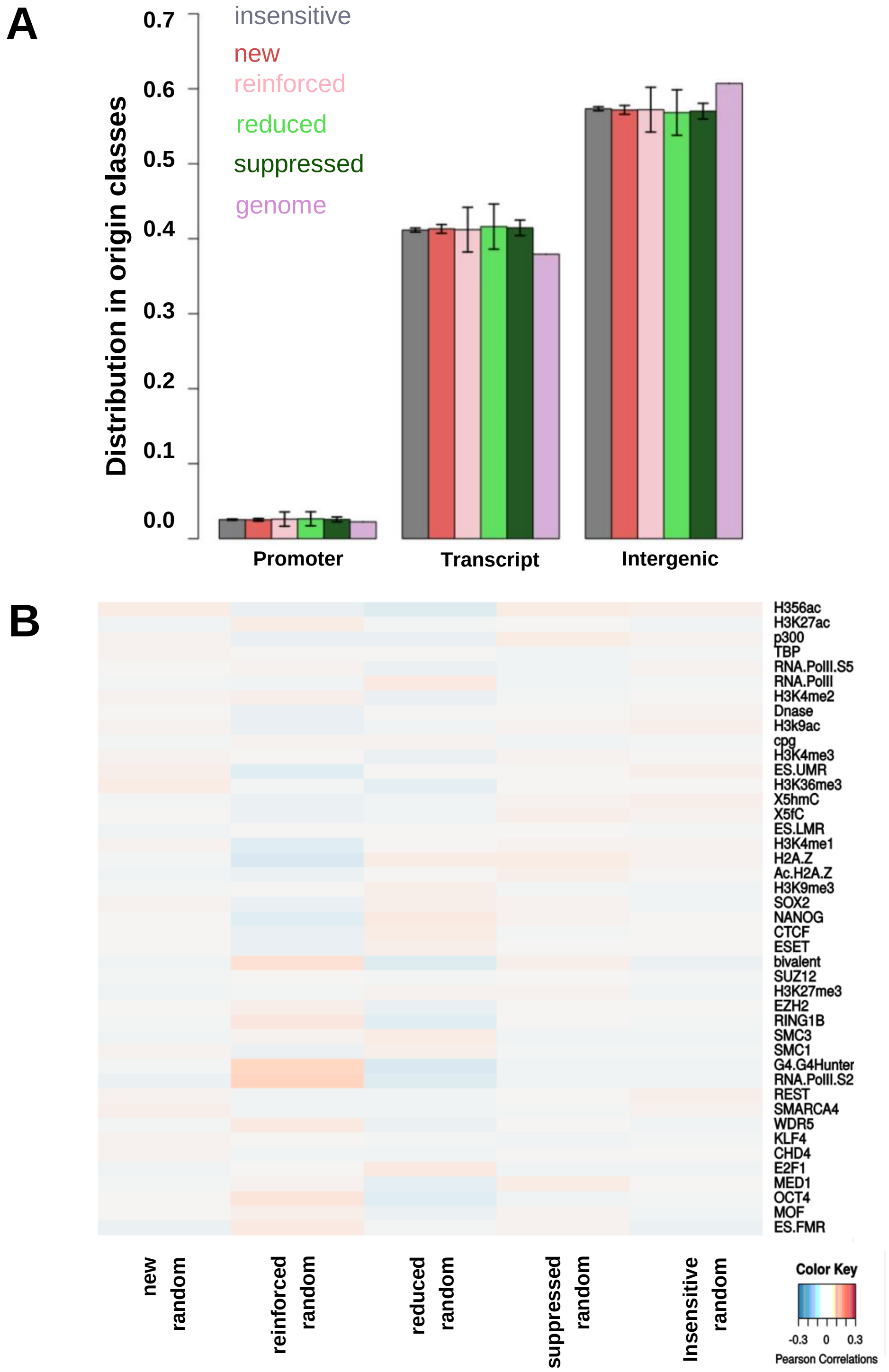

Supplementary Figure 6. Association of randomized origins with transcription-related elements and transcription activity measured by RNA-seq.

A) To be compared with Fig. 5B. Association of randomized origins with promoters, transcribed genes, and intergenic regions. Randomization was done by shuffling the IS coordinates, while keeping the chromosomal distribution of origins in each class. The value reported is the mean for 1000 shuffling events and the error bar represents the corresponding standard deviation.

B) Epigenetic mark distribution in randomized replication origins. To be compared with Figure 5C.

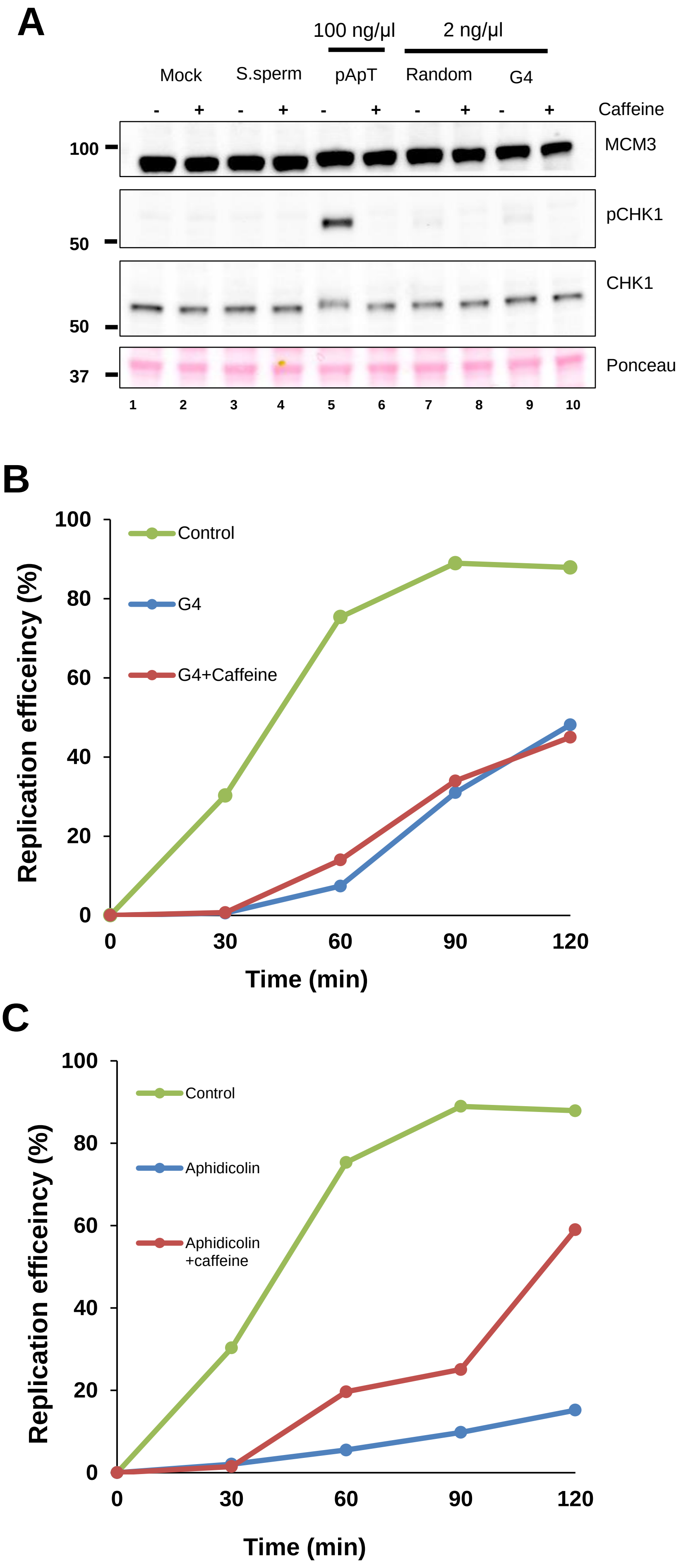
A
100 ng/µl
2 ng/µl
Mock
S.sperm
pApT
Random
G4
- + - + - + - + - +
Caffeine
100
MCM3
pCHK1
50
CHK1
50
Ponceau
37
1 2 3 4 5 6 7 8 9 10
B
Control
G4
G4+Caffeine
Replication efficeincy (%)
0 20 40 60 80 100
0 30 60 90 120
Time (min)
C
Control
Aphidicolin
Aphidicolin
+caffeine
Replication efficeincy (%)
0 20 40 60 80 100
0 30 60 90 120
Time (min)

Supplementary Figure 7. OGRE/G4 oligonucleotides do not induce a checkpoint response in *X. laevis* egg extracts.

A) Competing oligonucleotides were incubated in *X. laevis* egg extracts at the same concentration as the ones used in the study. The checkpoint response was analyzed by western blotting using anti-phosphorylated CHK1 and pCHK1 antibodies. pApT oligonucleotides at high concentration were used as positive control. CHK1 phosphorylation was sensitive to the ATR/ATM kinase inhibitor caffeine.

B) Time-course analysis of replication efficiency after addition of sperm nuclei to LSEs pre-incubated with H20 (mock), G4-forming oligonucleotides (G4) or G4-oligonucleotides and Caffeine (G4 + caffeine). Caffeine overcomes phosphorylated CHK1-dependent inhibition of DNA replication, but does not restore replication of sperm nuclei in the presence of ds OGRE/G4 oligonucleotides.

C) Time-course analysis of replication efficiency after addition of sperm nuclei to LSEs pre-incubated with H20 (mock), Aphidicolin (Aphidicolin) or Aphidicolin and caffeine (Aphidicolin + caffeine). Aphidicolin induces checkpoint activation and inhibition of DNA replication that can be restored by addition of caffeine.

Name	Chr	Sequence	Relation to transcription	Timing	G4H score	G4 formation
Ori1	11	GGGGGCGGGGAGGGAAGGGGG	1st Intron	Early	3.05	Yes
Ori2	11	GGGGGATGGGGTTGGAATGGGGGCGGG	1st Intron	Early	2.52	Yes
Ori ins A	19	TGGTGGGGGTGGGGGTAGGGGTGGGGGGAGA	1st Intron	Early	2.93	Yes
Ori ins B	16	AGGGGGATGGGAGGGGGAATGGGGGC	Intron	Late	2.88	Yes
Ori ins C	13	AGGGGGAGTGGGAGTGTAGGGGATCGGGGGGGGGGGTGTGAGTATGGGGGA	Intergenic	Late	2.33	Yes
Ori ins D	1	AGGGAGAGGGAGGATGGGATAGGGGGT	Intergenic	Late	2.21	Yes
Ori ins E	10	TGGTAGGGGTGGGGTGGGGGCTGGGGC	Intergenic	Early	2.84	Yes
Ori ins F	3	CAAAGGGCAGGCGGGGGTGGGGGGGTGGGGGTGGGGGCTCATGCAG	Intron	Early	2.51	Yes
Ori ins G	15	AGGGGGAGTGGGTGGGTAGGGGATTGGGGGGGT	Intergenic	Early	2.68	Yes
Ori new A	5	AGGGTGGGTGGGTGGGTGGGTGGGTGGGGC	Intergenic	Early	2.50	Yes
Ori new B	15	TGGCGGGGCGGGGGGGGGGTGCGGGGGAGGT	Intron	Early	2.83	Yes
Ori new C	4	AGGAGGGAGGGAGGGAGGGAGGGA	Intergenic	Late	2.23	Yes
Ori new D	13	TGGGGGTGGGTGGGTATGGGGGA	Intron	Late	2.76	Yes
Ori new E	5	CGGGGGGGATGGGGGGTCCTCAGGGGGCAGGGA	Intergenic	Early	2.42	Yes
Ori new F	1	CGGTGTGGGGTGGGGTGGGGTGGGGGAGA	Intergenic	Early	2.74	Yes
Ori new G	3	TGGGCTGGGGGCTGGAGGGGGGGATACTGGGA	Intergenic	Early	2.23	Yes
Ori new H	10	AGGGGGGACTGGGAGAGGGGAGAAGGA	Intron	Early	2.38	Yes
Ori new I	17	TGGCATGGGGGTCCGGGGGAGGGT	Intron	Early	2.18	Yes
22ag		AGGGTTAGGTTAGGGTTAGGG			1.48	NA
1XAV		TGAGGGTGGGTAGGGTGGGTAA			1.68	NA
ds26		CAATCGGATCGATCCGATTG			0	No
dT30		TTTTTTTTTTTTTTTTTTTTTTTTTTTTTT			0	No

Supplementary Table 1: OGRE/G4 sequences associated with origins and tested in this study.

Ori1 and Ori2 were used for genetic experiments in mouse cells and episomal vector replication. Putative G4-forming sequences found in the vicinity of insensitive (ins) and new (new) origins after PhenDC3 treatment are also indicated. The potential of each sequence to form a G4 was predicted by the G4-Hunter software (G4H score) and by testing them using two spectroscopic techniques (thermal difference spectrum and circular dichroism).

Oligo name	Sequence
gRNA insertion F	ACACCGATTTCTCAGTGTTAATTTTG
gRNA insertion R	AAAACAAAATTAACACTGAGAAATCG
SURV_C_S697	TCCATGGGGATCTCCTGAGG
SURV_C_AS697	CAAAAACACCAGAAGGAACACAG
Ori1 C1	CGC AGC AGT TAG GTT GTG CTG
C3 AS1	GAA AGG TTC TGA TTG GCT GGG
Ori1 G1	ATG CAG CCC ACC CTT TCA ATT C
pBS1529S	CAA CCC GGT AAG ACA CGA CT
pBS1726AS	CCG GAT CAA GAG CTA CCA AC
Bcgd1 F	AGA AGC CCT TGA CCA TGA GA
Bcgd1 R	AGG TGG CAG AAT GCT AAT GC
Bcgd2 F	CGG CAT GAT TTG GAC GTA A
Bcgd2 R	AGCAACAGGCAAGGTATTCAACA
Ori1 primer 1 F	GAATTGAAAGGGTGGGCTGC
Ori1 primer 1 R	TTGGACCTTTGAGGCTGGAC
Ori1 primer 2 F	CTT GTT TGT GAC TGG GCT GA
Ori1 primer 2 R	CAC TGC TTG TTC GCA CCT TA
Ori1 primer 3 F	GGACTGAGTGAT GCA GTGGT
Ori1 primer 3 R	ACA GGC GCA ATG TCCATA CT
Ori2 F	GGG CAC TTC AAA GCA ATG TT
Ori2 R	CCG TTT CTG GTG TTG AGG AT
5'junction F	CTG AAG ATT CTC AAG AGC GGC
5'junction R	CGC GAT CCT TGT TGG AGT TG
3'junction F	TGC TAG CCA GCC CTT TCT AA
3'junction R	TTT CAG AGG GTC CTC AAA GC
gRNA Ori1 delG4 1F	ACACCGCCTAAAGACCGAGTGGGGGG
gRNA Ori1 delG4 1R	AAAACCCCCCACTCGGTCTTTAGGCG
gRNA Ori1 delG4 2F	ACACCGGAACCTCGCAGCAGTGACCG
gRNA Ori1 delG4 2R	AAAACGGTCACTGCTGCGAGGTTCCG
Ori1 742 F	ACAATCGCACACAAACACCAC
Ori1 742 R	AGCGCTCACACGATTCCTT
Ori1 742 F	ACAATCGCACACAAACACCAC
Ori1 742 R	AGCGCTCACACGATTCCTT
Rai1c4ex3F (Rai1 a)	AGG AAC ACT GGG TCC ATG AG
Rai1c4ex4R (Rai1 a)	GCA CAT GGG TAG TGG TAG GT
Rai1qPCR130F (Rai1 b)	ATG CAG TCT TTT CGA GAA AGG TG
Rai1qPCR130R (Rai1 b)	GCT GCC GAT CAC AGC TTA G
Gapdh ex4-F	GTGCTGAGTATGTCGTGGAG
Gapdh ex5-R	GAGATGATGACCCTTTTGGCTC
b-actin-ex2-fw (actin 1)	GAACCCTAAGGCCAACCGTG
b-actin-ex3-rv (actin 1)	GGAGTCCATCACAATGCCTG
b-actinRT F1 (actin 2)	CGCCACCAGTTCGCCATGGA
b-actinRT R1 (actin 2)	TACAGCCCGGGGAGCATCGT
b-actinRT F1 (actin 2)	CGCCACCAGTTCGCCATGGA
b-actinRT R1 (actin 2)	TACAGCCCGGGGAGCATCGT

Supplementary Table 2. Sequences of the oligonucleotides used in the study.

Name	G4H score (sequence length [nt])	5'Fam-Sequence-Tamra 3'	Structure	Delta Tm (°C) PhenDC3 (1 µM)
F21T	1.71 (21)	**GGG** TTA **GGG** TTA **GGG** TTA **GGG**	G4 polymorphic	29.1
FKit2T	2.1 (22)	**GGG** C **GGG** CGCGA **GGG** AG **GGG**	G4 polymorphic	17.5
FHIV32T	1.21 (24)	CA **GGG** A **GG** C**G**T **GG** CCT **GGG** C **GGG** A	G4 Hybrid	23.9
FHIV321T	1.19 (21)	TT **GG** CCT **GGG** C **GGG** ACT **GGG** A	G4 Antiparallel	26.3
F21RT	1.71 (21)	**GGG** UUA **GGG** UUA **GGG** UUA **GGG**	G4 Antiparallel	30.1
F21KrasRT	1.52 (21)	A **GGG** C **GG** T**G**T **GGG** AA**G**A **GGG** A	G4 Antiparallel	16.1
F21CTAT	1.57 (21)	**GGG** CTA **GGG** CTA **GGG** CTA **GGG**	G4 Antiparallel	16.5
FdxT	0 (21)	TAT A**G**C TAT A-**hexa ethylene glycol** -T ATA<br>**G**CT ATA	dsDNA. Hairpin	0.2

Supplementary Table 3. Sequences used for the FRET melting assay.

FRET melting assay measures the affinity of G4 stabilizers for the tested sequences that are predicted to form G4 structures. The column “Structure“ indicates the structure adopted by the corresponding oligonucleotide, which is either a hairpin duplex (for FdxT) or a quadruplex (G4) with different possible topologies: parallel, antiparallel, hybrid, or a mixture (« polymorphic »). As shown in the rightmost column (delta Tm) PhenDC3 stabilizes all G4 structures (delta Tm > +16°C for all G4) and can therefore be considered as a G4 ligand having no strong preference for one given conformation. It is clearly specific for G4 over duplexes though, as the delta Tm for FdxT is close to zero.

(+/- 500bp from center)	total	QP+	QP-	G4H+	G4H-	%QP	%G4H
**insensitive**	34593	23512	11081	25605	8988	68.0	74.0
**new**	6972	5171	1801	5284	1688	74.2	75.8
**reinforced**	279	202	77	192	87	72.4	68.8
**reduced**	291	172	119	176	115	59.1	60.5
**suppressed**	2264	436	1828	369	1895	19.3	16.3
**total**	44399	29493	14906	31626	12773	66.4	71.2
**all control**	37427	24322	13105	26342	11085	65.0	70.4
**all PhenDC3**	42135	29057	13078	31257	10878	69.0	74.2
**oris changed**	9806	5981	3825	6021	3785	61.0	61.4

Supplementary Table 4: Origin association with OGRE/G4 according to G4-Hunter (G4H) and Quadparser (QP).
The number of origins found in each class, and their classification in OGRE/G4-associated (+) and -independent (-) origins, according to the QP and G4H putative G4 maps. Origins were considered to be associated with an OGRE/G4 motif when they contained at least one OGRE/G4 sequence upstream the IS at a maximal distance of 500 bp. There was no significant change in OGRE/G4-association between G4-predicting tools.

Mark/factor	Year	PMID	GEO_GSE
H3K56ac	2013	23798425	GSE47387_GSM1148636
H3K27ac	2012	encodeproject.org	GSE31039_GSM1000126
p300	2013	23602153	GSE41545_GSM1019072
TBP	2010	20720539	GSE22562
RNA PolII S5P	2010	20434984	GSE20530_GSM515662
RNA PolII	2010	20434984	GSE20530_GSM515667
H3K4me2	2013	23028048	GSE18515_GSM461266
DHS (Dnase)	2013	encodeproject.org	GSE37074_GSM1014154
H3K9ac	2012	22920947	GSE31284_GSM775313
CpGi	2015	25428374	
H3K4me3	2013	23352811	GSE38164_GSM936113
ES UMR	2013	22170606	GSE30202
H3K36me3	2012	encodeproject.org	GSE31039_GSM1000125
5hmC	2013	23602153	GSE41545_GSM857136
5fC	2013	23602153	GSE41545_GSM986287
ES LMR	2013	22170606	GSE30202
H3K4me1	2012	encodeproject.org	GSE31039_GSM1000121
H2A.Z	2013	23260488	GSE34483_GSM849928
Ac H2A.Z	2013	23260488	GSE34483_GSM849929
H3K9me3	2012	encodeproject.org	GSE31039_GSM1000147
SOX2	2008	18555785	GSE11431_GSM288347
Nanog	2008	18555785	GSE11431_GSM288345
CTCF	2008	18555785	GSE11431_GSM288351
ESET	2009	19884257	GSE17642_GSM440256
bivalent_domain	2007	17603471	GSE12241
SUZ12	2012	22438827	GSE28325_GSM700554
H3K27me3	2013	23104054	GSE41589_GSM1019772
EZH2	2009	20064375	GSE18776_GSM480161
RNF2(ring1B)	2012	22325148	GSE23716_GSM585229
SMC3	2010	20720539	GSE22562_GSM560343
SMC1	2010	20720539	GSE22562_GSM560341
G4	2016	26792894	G4Hunter
RNA PolII S2P	2010	20434984	GSE20530_GSM515663
REST	2012	22396653	GSE48122_GSM1169011
SMARC4	2009	19279218	GSE14344_GSM359413
WDR5	2011	21477851	GSE22934_GSM566279
KLF4	2008	18555785	GSE11431_GSM288354
CHD4	2012	22297846	GSE27841_GSM687284
E2F1	2008	18555785	GSE11431_GSM288349
MED1	2010	20720539	GSE22562_GSM560347
OCT4	2012	22608532	GSE36388_GSM892276
MOF	2012	22862943	GSE37268_GSM915227/8
ES FMR	2013	22170606	GSE30202

Supplementary Table 5. Reference data for the epigenetic marks.

Name	GC content	Sequence	Reverse complement
OGRE/G4-containing oligonucleotide,	71.3 %	CGAGGTTCCTAGGCGCCTAAAGAC<br>CGAGTGGGGGCGGGGAGGGAAG<br>GGGGTGCTGTGCGTGCGCGCGCGC<br>GTGCCAAAGC	GCTTTGGCACGCGCGCG<br>CGCACGCACAGCACCCC<br>CTTCCCTCCCCGCCCCCA<br>CTCGGTCTTTAGGCGCCT<br>AGGAACCTCG
Random oligonucleotide 1	47.5 %	TTCTGCGTTACTTTAACCGCGACGG<br>ACGCCTGAGCTTTGACCTAATTCGC<br>AGGGTGGGATATTGCGTTTTTAATT<br>GAGGT	ACCTCAATTAAAAACGC<br>AATATCCCACCCTGCGA<br>ATTAGGTCAAAGCTCAG<br>GCGTCCGTCGCGGTTAA<br>AGTAACGCAGAA
Random oligonucleotide 2	48.8 %	GCAAGTCAGCAGCATTACCACTGT<br>CTCCTGTTGTCAGAATAACCGGACA<br>GCCTGGGTTTCAAAGTGGAGCTGT<br>GTATGCT	AGCATACACAGCTCCAC<br>TTTGAAACCCAGGCTGT<br>CCGGTTATTCTGACAACA<br>GGAGACAGTGGTAATGC<br>TGCTGACTTGC
AT-rich (70%) Oligonucletide	30 %	TGCTAAATCAATTGTTATCGGTTCT<br>TAAAACAATCGCTCACAAAACGGA<br>TTAAACAAAGAAAAGCAAATATTG<br>TCAATGA	TCATTGACAATATTTGCT<br>TTTCTTTGTTTAATCCGTT<br>TTGTGAGCGATTGTTTTA<br>AGAACCGATAACAATTG<br>ATTTAGC

Supplementary Table 6. G-rich sequences tested in the oligonucleotide competition assays in *X. laevis* egg extracts. Random and AT-rich oligonucleotides were designed (http://mkwak.org/oligorand/):

Antibody	Company	Reference	Dilution
Study in mouse cells			
Anti-OCT4	Abcam	ab19857	1/500
Anti-pCHK1	Cell Signaling	2341T	1/250
Anti-CHK1	Santa Cruz	sc-8408	1/500
Anti-Actin	Sigma	A4700	1/500
secondary HRP-linked Anti-Mouse IgG	GE Healthcare	NA931V	1/4000
secondary HRP-linked Anti-Rabbit IgG	GE Healthcare	NA934V	1/4000
Study in *X. laevis* egg extracts			
Anti-ELYS	laboratory stock	PMID: 17825564, PMID: 17235358	1/500
Anti-CDC45	laboratory stock	PMID: 12384698	1/1000
Anti-PCNA	Sigma	P8825	1/2500
Anti-H3	Abcam	Ab1791	1/2000
Anti-MCM4	laboratory stock	PMID: 12384698	1/1000
Anti-CDC6	laboratory stock	PMID: 12384698	1/500
Anti-ORC5	laboratory stock	PMID: 31160578	1/1000
Anti-H2B	Abcam	ab1790	1/2000
Anti-MCM3	laboratory stock	PMID: 15707891	1/2000
Anti-RPA34	laboratory stock	PMID: 15456845	1/500
Anti-CHK1	Santa Cruz	sc-8404	1/250
Anti-pCHK1	Cell Signaling	2341S	1/500

Supplementary Table 7. References of antibodies used in this study.
