# Supplementary material for: Involvement of G-quadruplex regions in mammalian replication origin activity
Source: Nat Commun. 2019 Jul 22;10:3274. doi: 10.1038/s41467-019-11104-0 (PMC6646384; doi:10.1038/s41467-019-11104-0)
Supplement: Supplementary file 3 — Reporting Summary [file 41467_2019_11104_MOESM3_ESM.pdf]

nature research

Corresponding author(s): Marcel Mechali

Last updated by author(s): Apr 29, 2019

Reporting Summary

Nature Research wishes to improve the reproducibility of the work that we publish. This form provides structure for consistency and transparency in reporting. For further information on Nature Research policies, see Authors & Referees and the Editorial Policy Checklist.

Statistics

For all statistical analyses, confirm that the following items are present in the figure legend, table legend, main text, or Methods section.

n/a	Confirmed	
☐	☒	The exact sample size (n) for each experimental group/condition, given as a discrete number and unit of measurement
☐	☒	A statement on whether measurements were taken from distinct samples or whether the same sample was measured repeatedly
☐	☒	The statistical test(s) used AND whether they are one- or two-sided<br>*Only common tests should be described solely by name; describe more complex techniques in the Methods section.*
☒	☐	A description of all covariates tested
☒	☐	A description of any assumptions or corrections, such as tests of normality and adjustment for multiple comparisons
☐	☒	A full description of the statistical parameters including central tendency (e.g. means) or other basic estimates (e.g. regression coefficient) AND variation (e.g. standard deviation) or associated estimates of uncertainty (e.g. confidence intervals)
☐	☒	For null hypothesis testing, the test statistic (e.g. F, t, r) with confidence intervals, effect sizes, degrees of freedom and P value noted<br>*Give P values as exact values whenever suitable.*
☒	☐	For Bayesian analysis, information on the choice of priors and Markov chain Monte Carlo settings
☒	☐	For hierarchical and complex designs, identification of the appropriate level for tests and full reporting of outcomes
☐	☒	Estimates of effect sizes (e.g. Cohen's d, Pearson's r), indicating how they were calculated

Our web collection on statistics for biologists contains articles on many of the points above.

Software and code

Policy information about availability of computer code

Data collection	Raw data collected form illumina sequencers. Information provided in the Data Availability section of the manuscript and NCBI GEO (GSE126477) [https://www.ncbi.nlm.nih.gov/geo/query/acc.cgi?acc=GSE126477]
Data analysis	Information provided in the Data Availability section of the manuscript, NCBI GEO (GSE126477) [https://www.ncbi.nlm.nih.gov/geo/query/acc.cgi?acc=GSE126477], [http://rsat-tagc.univ-mrs.fr/g4/g4_data.html] and [https://github.com/LacroixLaurent/G4Hunter_mm10_Ori] . In the data analyses we used bowtie, macs2, SICER, R, SnakeMake, DeSeq open source softwares

For manuscripts utilizing custom algorithms or software that are central to the research but not yet described in published literature, software must be made available to editors/reviewers. We strongly encourage code deposition in a community repository (e.g. GitHub). See the Nature Research guidelines for submitting code & software for further information.

Data

Policy information about availability of data

All manuscripts must include a data availability statement. This statement should provide the following information, where applicable:

- Accession codes, unique identifiers, or web links for publicly available datasets
- A list of figures that have associated raw data
- A description of any restrictions on data availability

Data supporting the findings of this study are available within the article and its supplementary information files, including uncropped scans of the most important blots. All data are available from the corresponding authors upon reasonable request. Data is available through GEO ID : GSE126477, and through our web companion site : http://rsat-tagc.univ-mrs.fr/g4/g4_data.html

Field-specific reporting

Please select the one below that is the best fit for your research. If you are not sure, read the appropriate sections before making your selection.

☒ Life sciences ☐ Behavioural & social sciences ☐ Ecological, evolutionary & environmental sciences

For a reference copy of the document with all sections, see nature.com/documents/nr-reporting-summary-flat.pdf

Life sciences study design

All studies must disclose on these points even when the disclosure is negative.

Sample size	The sample size for the experimental data was precised in the manuscript.
Data exclusions	No data was excluded.
Replication	All attempts at replication were successfull.
Randomization	When it applies, randomization method is described in Methods and Supplementary Methods section:
Blinding	Investigators were not blinded to allocation during experiments and outcome assessment.

Reporting for specific materials, systems and methods

We require information from authors about some types of materials, experimental systems and methods used in many studies. Here, indicate whether each material, system or method listed is relevant to your study. If you are not sure if a list item applies to your research, read the appropriate section before selecting a response.

Materials & experimental systems

n/a	Involved in the study	
☐	☒	Antibodies
☐	☒	Eukaryotic cell lines
☒	☐	Palaeontology
☒	☐	Animals and other organisms
☒	☐	Human research participants
☒	☐	Clinical data

Methods

n/a	Involved in the study	
☒	☐	ChIP-seq
☐	☒	Flow cytometry
☒	☐	MRI-based neuroimaging

Antibodies

Antibodies used	H3 (Abcam, ab1791, dilution 1/2000), H2B (Abcam, ab1790, dilution 1/2000), phosphorylated CHK1 (Cell Signaling, 2341S, dilution 1/250), PCNA (Sigma, P8825, dilution 1/2500), RPA34 (dilution 1/500), MCM3 (dilution 1/2000), CDC45 (dilution 1/1000), ELYS (dilution 1/500), MCM4 (dilution 1/1000), anti-Chk1 (dilution 1/500), anti-ORC5 (dilution 1/1000), anti-CDC6 (dilution 1/500), OCT4 (Abcam, ab19857, dilution 1/500), actin (Sigma, A4700, dilution 1/500), HRP-linked ECL anti-mouse IgG (GE Healthcare, NA931V, dilution 1/4000), HRP-linked ECL anti-rabbit IgG (GE Healthcare, NA934V, dilution 1/4000)
Validation	All the antibodies used were validated by the manufacturer and checked by us by Western blot.<br>Validation data are provided on the manufacturer's website, along with citation and references.

Eukaryotic cell lines

Policy information about cell lines

Cell line source(s)	CGR8 mouse ES cells obtained from Austin Smith's laboratory, Department of Biochemistry, University of Cambridge, UK<br>NIH/3T3 cells purchased from ATCC CRL-1658
Authentication	The cell lines used were previously authenticated by the providers.
Mycoplasma contamination	All cell lines were regularly tested for mycoplasma contamination.
Commonly misidentified lines<br>(See ICLAC register)	N/A

Flow Cytometry

Plots

Confirm that:

☒ The axis labels state the marker and fluorochrome used (e.g. CD4-FITC).

☒ The axis scales are clearly visible. Include numbers along axes only for bottom left plot of group (a 'group' is an analysis of identical markers).

☒ All plots are contour plots with outliers or pseudocolor plots.

☐ A numerical value for number of cells or percentage (with statistics) is provided.

Methodology

Sample preparation	For cell cycle analysis, cells were incubated 10 min in BrdU containing medium (10 µM BrdU). After ethanol-fixation cells were incubated with RNase A and propidium iodide. BrdU incorporation was detected (after HCl denaturation) using a mouse anti-BrdU antibody followed by an anti-mouse FITC-coupled antibody. BrdU incorporation was quantified by measuring the fluorescence intensity of BrdU-positive cells using a MACSQuant cytometer (Miltenyi).
Instrument	MACSQuant cytometer (Miltenyi)
Software	MACSQuantify (version 2.6).
Cell population abundance	No cell sorting was performed.
Gating strategy	N/A

☐ Tick this box to confirm that a figure exemplifying the gating strategy is provided in the Supplementary Information.
