# Supplementary material for: Involvement of G-quadruplex regions in mammalian replication origin activity
Source: Nat Commun. 2019 Jul 22;10:3274. doi: 10.1038/s41467-019-11104-0 (PMC6646384; doi:10.1038/s41467-019-11104-0)
Supplement: Supplementary file 4 — Source Data [file 41467_2019_11104_MOESM4_ESM.zip › Source Data - Uncropped Gels.pdf]

Fig 6D

Uncropped blot

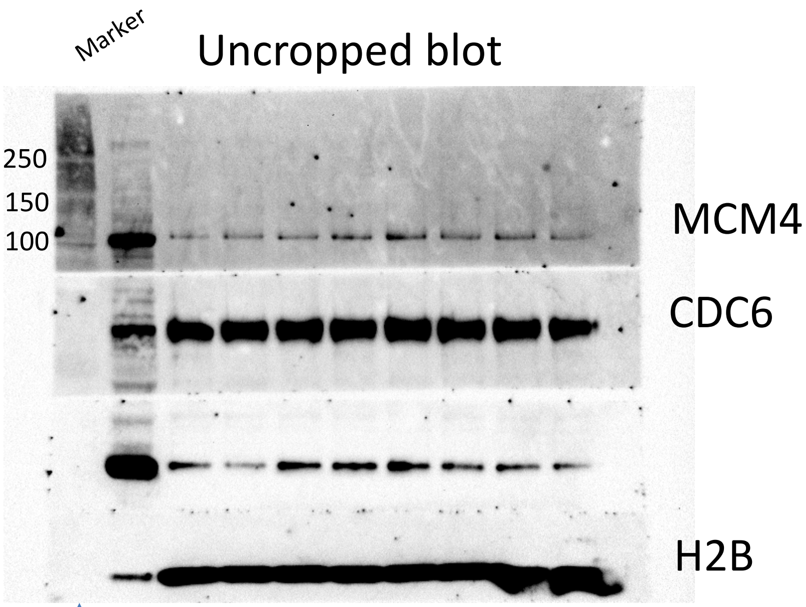

Marker –high molecular weight proteins of ladder can be weakly seen after on photographic films upon development due to weak unspecific antibody binding

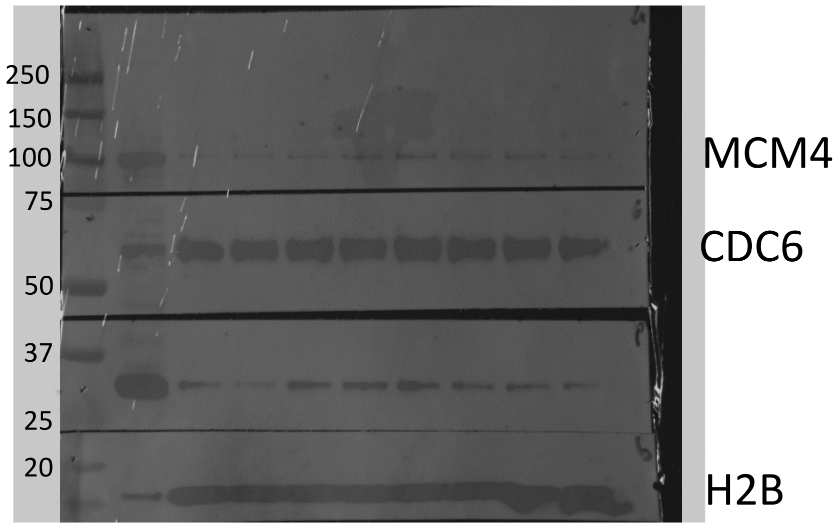

Uncropped ladder visible on membranes

Membranes overlaid with developed films after exposure to protein-antibody complexes on the western blot membrane: permits to align the corresponding MW markers

Fig 6D

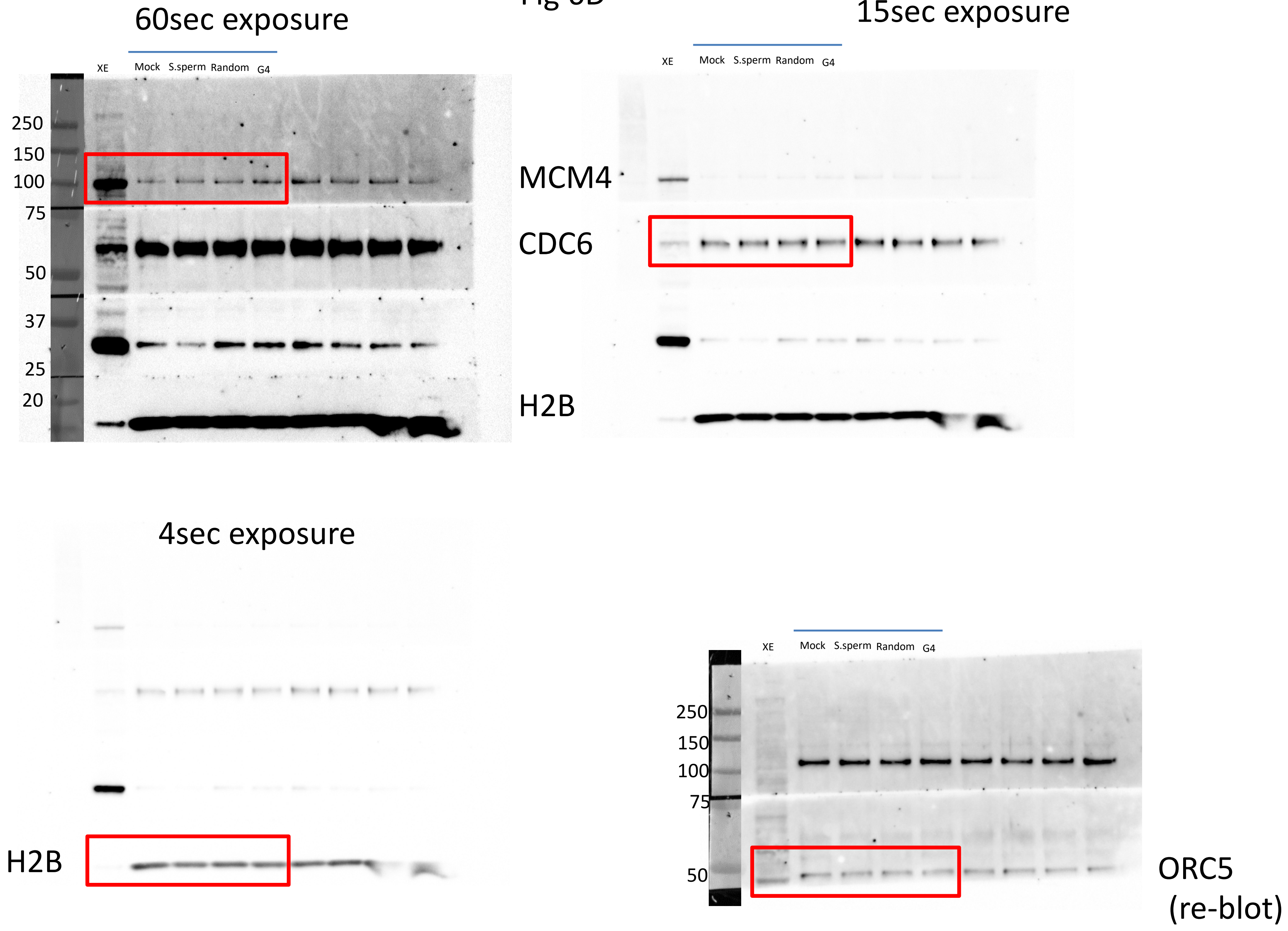
60sec exposure
XE
Mock
S.sperm
Random
G4
250
150
100
75
50
37
25
20
MCM4
CDC6
H2B
15sec exposure
4sec exposure
H2B
ORC5
(re-blot)

Fig SI 7A

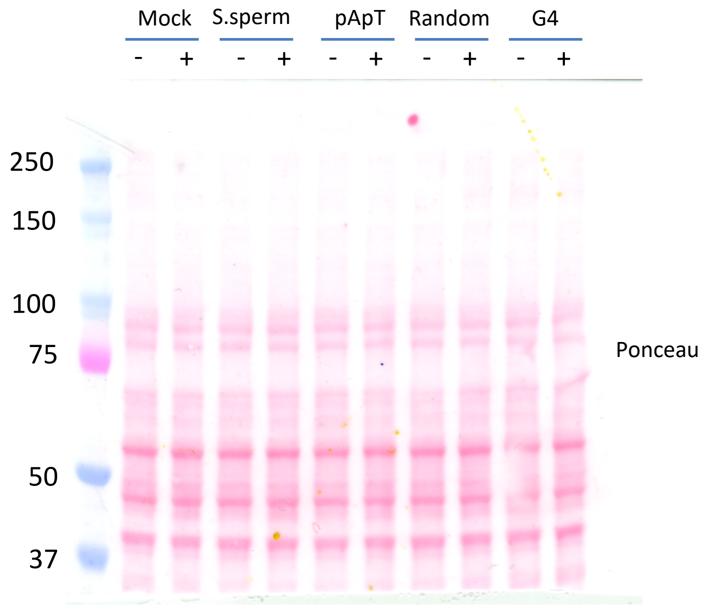

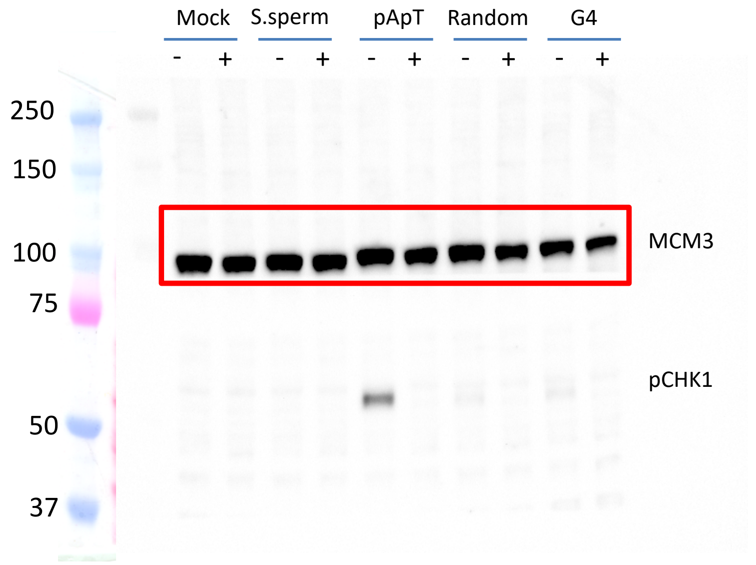

Uncropped blot

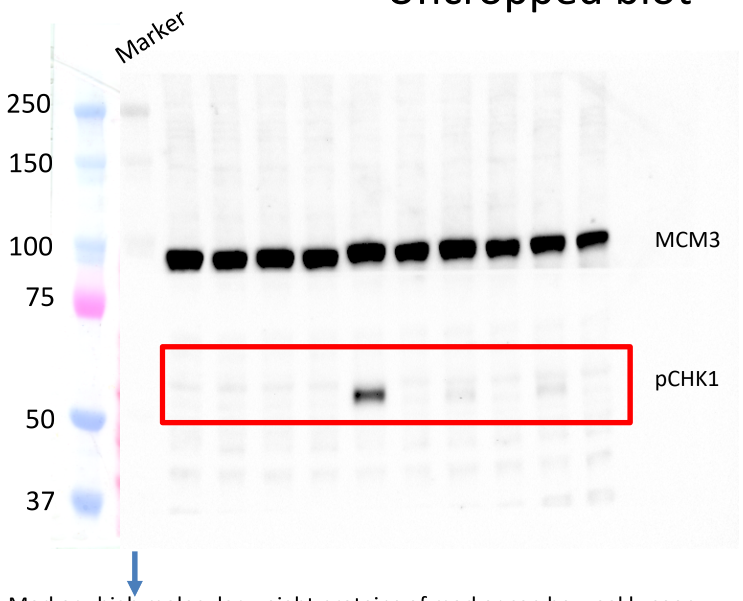

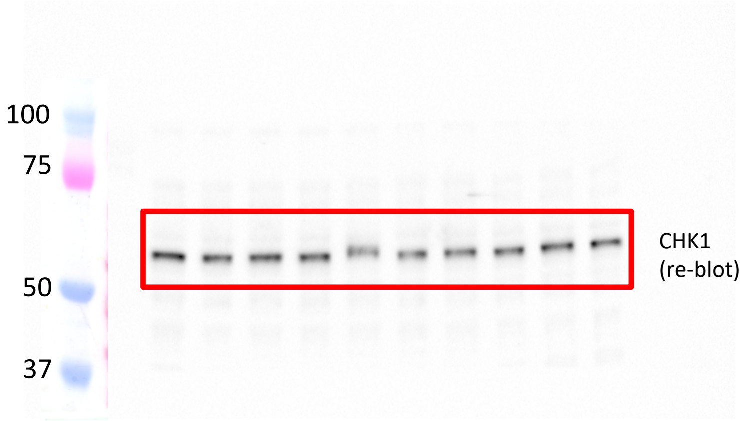

Marker –high molecular weight proteins of marker can be weakly seen after on photographic films upon development due to weak unspecific antibody binding

Fig SI 3A

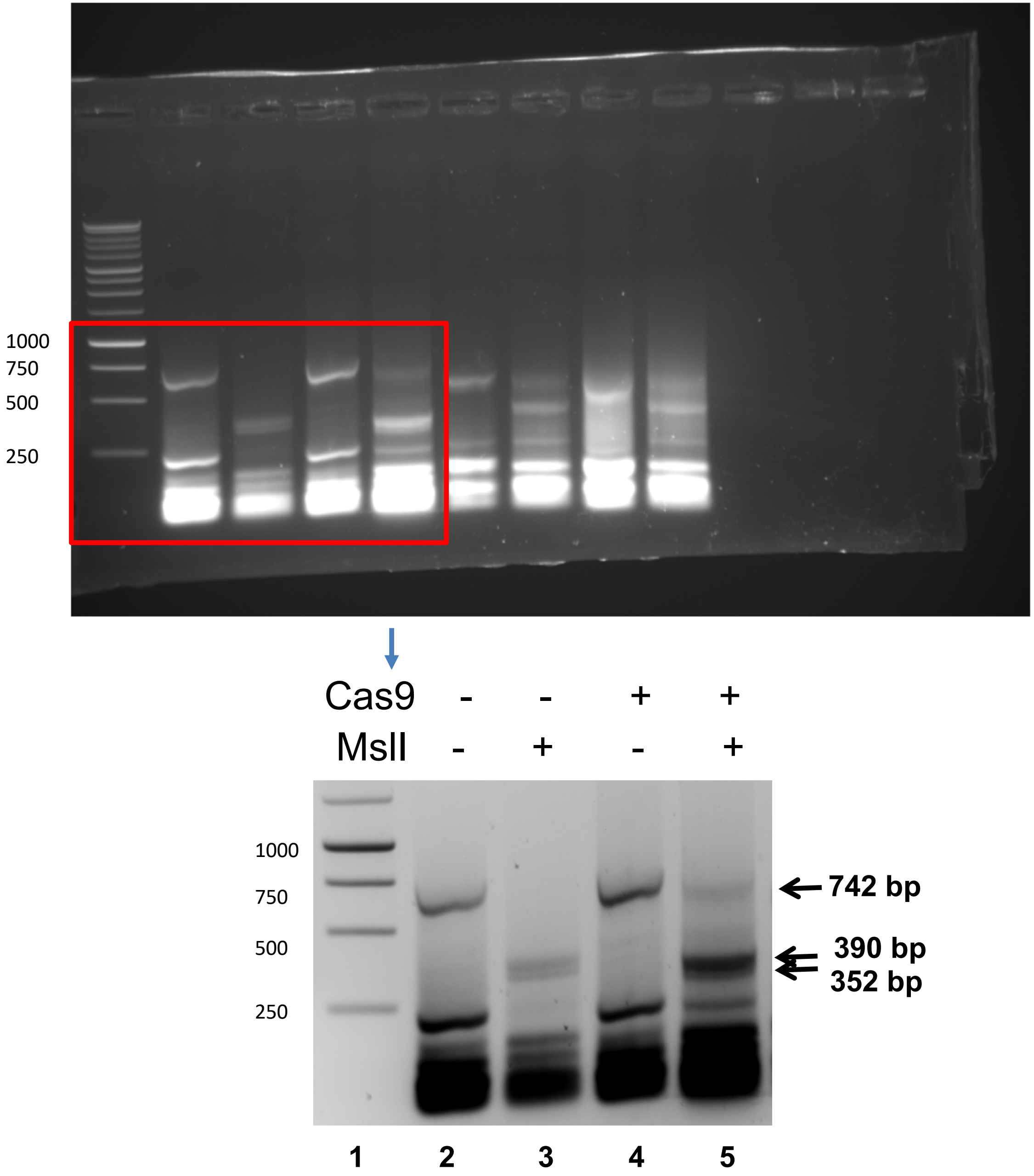
1000
750
500
250
Cas9 - - + +
MslI - + - +
1000
750
500
250
742 bp
390 bp
352 bp
1 2 3 4 5
